# Orphan Genes Shared by Pathogenic Genomes Are More Associated with Bacterial Pathogenicity

**DOI:** 10.1128/mSystems.00290-18

**Published:** 2019-02-12

**Authors:** Sarah Entwistle, Xueqiong Li, Yanbin Yin

**Affiliations:** a Department of Biological Sciences, Northern Illinois University, DeKalb, Illinois, USA; b Department of Food Science and Technology, University of Nebraska—Lincoln, Lincoln, Nebraska, USA; University of Delhi

**Keywords:** ORFan, orphan gene, horizontal gene transfer, pathogenic island, pathogenicity, prophage, virulence factor

## Abstract

Orphan genes (also known as ORFans [i.e., orphan open reading frames]) are new genes that enable an organism to adapt to its specific living environment. Our focus in this study is to compare ORFans between pathogens (P) and nonpathogens (NP) of the same genus. Using the pangenome idea, we have identified 130,169 ORFans in nine bacterial genera (505 genomes) and classified these ORFans into four groups: (i) SS-ORFans (P), which are only found in a single pathogenic genome; (ii) SS-ORFans (NP), which are only found in a single nonpathogenic genome; (iii) PS-ORFans (P), which are found in multiple pathogenic genomes; and (iv) NS-ORFans (NP), which are found in multiple nonpathogenic genomes. Within the same genus, pathogens do not always have more genes, more ORFans, or more pathogenicity-related genes (PRGs)—including prophages, pathogenicity islands (PAIs), virulence factors (VFs), and horizontal gene transfers (HGTs)—than nonpathogens. Interestingly, in pathogens of the nine genera, the percentages of PS-ORFans are consistently higher than those of SS-ORFans, which is not true in nonpathogens. Similarly, in pathogens of the nine genera, the percentages of PS-ORFans matching the four types of PRGs are also always higher than those of SS-ORFans, but this is not true in nonpathogens. All of these findings suggest the greater importance of PS-ORFans for bacterial pathogenicity.

**IMPORTANCE** Recent pangenome analyses of numerous bacterial species have suggested that each genome of a single species may have a significant fraction of its gene content unique or shared by a very few genomes (i.e., ORFans). We selected nine bacterial genera, each containing at least five pathogenic and five nonpathogenic genomes, to compare their ORFans in relation to pathogenicity-related genes. Pathogens in these genera are known to cause a number of common and devastating human diseases such as pneumonia, diphtheria, melioidosis, and tuberculosis. Thus, they are worthy of in-depth systems microbiology investigations, including the comparative study of ORFans between pathogens and nonpathogens. We provide direct evidence to suggest that ORFans shared by more pathogens are more associated with pathogenicity-related genes and thus are more important targets for development of new diagnostic markers or therapeutic drugs for bacterial infectious diseases.

## Abstract (precis)

Recent pangenome analyses of numerous bacterial species have suggested that each genome of a single species may have a significant fraction of its gene content unique or shared by a very few genomes (i.e., ORFans). We selected nine bacterial genera, each containing at least five pathogenic and five nonpathogenic genomes, to compare their ORFans in relation to pathogenicity-related genes. Pathogens in these genera are known to cause a number of common and devastating human diseases such as pneumonia, diphtheria, melioidosis, and tuberculosis. Thus, they are worthy of in-depth systems microbiology investigations, including the comparative study of ORFans between pathogens and nonpathogens. We provide direct evidence to suggest that ORFans shared by more pathogens are more associated with pathogenicity-related genes and thus are more important targets for development of new diagnostic markers or therapeutic drugs for bacterial infectious diseases.

## INTRODUCTION

Orphan genes, also known as ORFans (i.e., orphan open reading frames), are new protein-coding genes restricted to taxonomically closely related genomes (1). ORFans are usually identified by a sequence similarity search against a protein sequence database such as the nonredundant (nr) protein database of NCBI (2, 3). We and others have shown that every newly sequenced genome contains a significant number of ORFans (4–6), although the percentages of ORFans vary considerably (5) in different species.

Earlier studies found that ORFans are shorter, have lower GC content, and evolve more rapidly (6–10). Therefore, ORFans were once thought to be mispredicted protein-coding genes. However, accumulating experimental evidence has been demonstrated that many ORFans correspond to real and functional proteins (7, 11–24). In addition, it has been suggested that newly evolved ORFan genes often confer new traits and play significant roles in assisting their host organisms to adapt to the ever-changing environments (5, 9). For example, an ORFan gene named *neaT* was characterized in extraintestinal pathogenic (ExPEC) Escherichia coli to have a key role in the virulence of ExPEC in zebrafish embryos (24). Therefore, although molecular biologists tend to focus more on conserved genes, the taxonomically restricted ORFans are likely to be more important for the emergence of species-specific traits: e.g., the ability of pathogens to infect their hosts.

Previously, ORFans have been shown to be enriched in genomic islands (GIs) of bacterial genomes (25). GIs are defined as horizontally transferred gene (HGT) clusters that often contain virulence factor (VF) genes and can transform nonpathogens to pathogens. Hence, many GIs are also known as pathogenicity islands (PAIs), a term we prefer to use in this article. In fact, PAIs were shown to contain more VF genes than the rest of the genome (26). Another study showed that 39% of ORFans in 119 prokaryotic genomes were found in clusters of genes with atypical base compositions (27), which correspond to horizontally transferred foreign elements from other bacteria or viruses. However, none of the previous large-scale analyses of prokaryotic ORFans (e.g., references 4, 28, 29, and 30) have distinguished pathogens and nonpathogens.

Recent pangenome analyses of numerous bacterial pathogens and their closely related nonpathogenic strains have suggested that each genome of a single species may have a significant fraction of unique gene content known as the variable genome (31–41). Many of the unique genes are lineage-specific ORFans; those unique genes residing in PAIs or prophages may have contributed to the bacterial pathogenicity (42, 43).

In this study, our goal was to study the association between ORFans and pathogenicity of bacteria by analyzing fully sequenced bacterial genomes, which have been classified into pathogen (P) and nonpathogen (NP) groups. We identified ORFans adopting the pangenome idea, according to which proteins from the variable genome are ORFans. Compared to previous studies, the novelty of this study is that we have classified ORFans into different groups: SS-ORFans (strain-specific ORFans present in just one genome), PS-ORFans (pathogen-specific ORFans shared by pathogenic genomes), and NS-ORFans (nonpathogen-specific ORFans shared by nonpathogenic genomes).

Specifically, using bacterial genomes from nine bacterial genera, we aimed to address the following questions by comparing genomes of the same genus. (i) Do pathogens have more genes than nonpathogens? (ii) Do pathogens have a higher percentage of ORFans than nonpathogens? (iii) Do pathogens have more pathogenicity-related genes (PRGs), such as genes in prophages and PAIs and genes identified as HGTs and VFs, than nonpathogens? (iv) Which group of ORFans is more represented in the four types of PRGs and thus is more likely to be associated with bacterial pathogenicity?

## RESULTS

### Overall comparisons of ORFans between pathogens and nonpathogens in nine genera.

The nine bacterial genera with more than five complete pathogenic genomes and five complete nonpathogenic genomes are shown in Table 1 (also see Materials and Methods). Here “complete” means that the genomes are fully determined and assembled. Bacteria of these genera are known to cause a number of common and devastating human diseases (see Table S1 in the supplemental material).

**TABLE 1:** Nine bacterial genera selected for the ORFan study

Genus	Phylum	No. of genomes			Range of:	
		Total	P	NP	No. of genes	Genome sizes (Mb)
*Bacillus*	Firmicutes	79	34	45	2,841–6,402	3.1–6.0
*Burkholderia*	Proteobacteria	33	27	6	4,248–8,006	3.0–4.4
*Clostridium*	*Firmicutes*	32	17	15	2,224–5,639	2.5–6.5
*Corynebacterium*	Actinobacteria	51	35	16	1,768–2,999	2.0–3.4
*Escherichia*	*Proteobacteria*	57	47	10	3,708–5,732	4.0–5.7
*Listeria*	*Firmicutes*	40	28	12	2,661–3,143	2.8–3.1
*Mycobacterium*	*Actinobacteria*	54	44	10	1,605–6,784	3.3–7.0
*Pseudomonas*	*Proteobacteria*	51	18	33	3,734–6,178	4.2–7.1
*Streptococcus*	*Firmicutes*	108	90	18	1,585–2,270	1.8–2.4

10.1128/mSystems.00290-18.1 TABLE S1 Statistics of diseases and bacteria. Download Table S1, XLSX file, 0.03 MB. Copyright © 2019 Entwistle et al. 2019 Entwistle et al. This content is distributed under the terms of the Creative Commons Attribution 4.0 International license.

**FIG 1:**
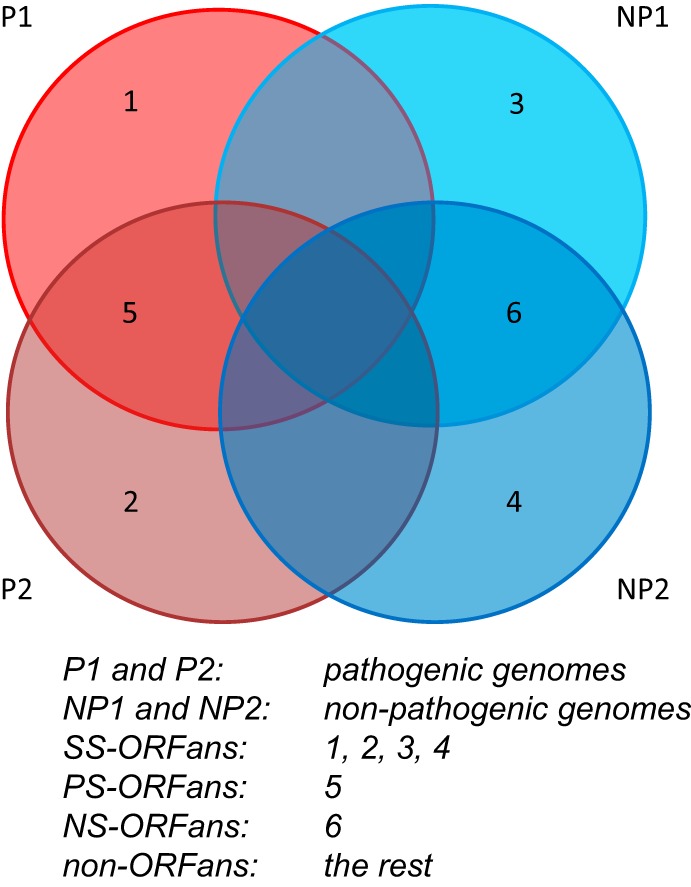
Pangenome idea to define different groups of ORFan genes and non-ORFan genes.

As shown in Table 2, the 505 genomes are grouped into 340 pathogenic (P) genomes (1,255,580 proteins) and 165 nonpathogenic (NP) genomes (657,172 proteins). The percentages of ORFans are calculated relative to the gene contents in the two groups of genomes, respectively (see Fig. 1 and Materials and Methods for how we defined the four groups of ORFans). In the 340 P genomes, the percentage of SS-ORFans is 1.39% and the percentage of PS-ORFans is 4.48%. Similarly, in the 165 NP genomes, the percentage of SS-ORFans is 2.60% and the percentage of NS-ORFans is 6.00%. Hence, the overall percentage of ORFans seems higher in NP than P genomes, which agrees with a previous study (19% nonpathogen-associated genes versus 14% pathogen-associated genes) (26).

**TABLE 2:** Comparisons of the four groups of ORFans in the P and NP genomes

Protein group	No. (%) of ORFans in^*a*^:	
	Total NP genomes	Total P genomes
All proteins		
SS-ORFans	17,081 (2.60)	17,455 (1.39)
PS-ORFans		56,196 (4.48)
NS-ORFans	39,437 (6.00)	
Non-ORFans	600,654 (91.40)	1,181,929 (94.13)
Prophage proteins		
SS-ORFans	1,459 (8.54)	2,138 (12.24)
PS-ORFans		10,539 (18.75)
NS-ORFans	3,747 (9.50)	
Non-ORFans	13,071 (2.18)	34,366 (2.91)
PAI proteins		
SS-ORFans	5,091 (29.81)	5,163 (29.58)
PS-ORFans		17,087 (30.41)
NS-ORFans	8,236 (20.88)	
Non-ORFans	37,412 (6.23)	84,006 (7.11)
VF proteins		
SS-ORFans	78 (0.46)	116 (0.66)
PS-ORFans		2,718 (4.84)
NS-ORFans	259 (0.66)	
Non-ORFans	109,216 (18.18)	210,988 (17.85)
HGT proteins		
SS-ORFans	5,486 (32.12)	4,694 (26.89)
PS-ORFans		13,857 (24.66)
NS-ORFans	15,587 (39.52)	

a The results shown represent 165 genomes and 657,172 proteins for total NP genomes and 340 genomes and 1,255,580 proteins for total P genomes.

Furthermore, Table 2 also shows the four groups of ORFans further broken into the four types of PRGs (pathogenicity-related genes [explained in Materials and Methods]). For example, the percentage of SS-ORFans in P genomes carried by prophages is 12.24%, which was calculated by no. of SS-ORFans in prophages/total no. of SS-ORFans: 2,138/17,455.

For prophages and PAIs, it is clear that ORFans of P genomes are more likely to be carried by PAIs and prophages than ORFans of NP genomes (e.g., for prophages, P genomes [18.75% + 12.24%] versus NP genomes [9.50% + 8.54%]). When looking at different ORFan groups, the percentage of PS-ORFans is always the highest (18.75% for prophages and 30.41% for PAIs). Additionally, it appears that ORFans are more likely to be carried by PAIs and prophages than non-ORFans in both P and NP genomes, which extends the finding made in reference 25.

For VFs, the numbers of ORFans annotated as VFs are very small, in contrast to much larger numbers for non-ORFans. Notably, 259 (0.66%) NS-ORFans are VFs, compared to 2,718 (4.84%) PS-ORFans being VFs. A previous study has shown that VFs are highly enriched in PAIs compared to non-PAI regions (26). Interestingly, here we showed that most VFs are found in non-ORFans (more conserved genes shared by P and NP genomes). This is likely because, as indicated in reference 26, there are VFs commonly found in P and NP genomes, which are more abundant in bacterial genomes than those pathogen-associated VFs.

For HGTs, non-ORFans were excluded in our HGT identification because they do not qualify, “having limited blastp hits in taxonomically close (genus-level) genomes” (see Materials and Methods). Table 2 shows that NP genomes have higher percentages of ORFans identified as HGTs than P genomes, contrary to the other three types of PRGs.

However, it should be noted that Table 2 combined ORFans of the nine genera as a whole for comparisons. Thus, the above observations could be biased due to the fact that some genera have more genomes (e.g., Streptococcus) or have better-annotated PRGs (e.g., Escherichia) than others. To obtain more statistically robust results without biases, we have counted the number of ORFans in each genome (see Data Set S1 in the supplemental material), calculated the percentages, and further statistically compared the P and NP genomes in each genus.

10.1128/mSystems.00290-18.5 DATA SET S1 The breakdown numbers of ORFans in each of 505 genomes grouped by genera. Download Data Set S1, XLSX file, 0.08 MB. Copyright © 2019 Entwistle et al. 2019 Entwistle et al. This content is distributed under the terms of the Creative Commons Attribution 4.0 International license.

### Pathogens do not always have more genes than nonpathogens.

The pairwise nonparametric Wilcoxon test *P* values (the second column of Table 3) show that not all genera have their P genomes carrying more genes than NP genomes. In four out of the nine genera: Bacillus, Escherichia, Pseudomonas, and Streptococcus, the P genomes have a higher number of genes than NP genomes. However, it is the opposite in three other genera: Clostridium, Corynebacterium, and Mycobacterium. This result remains the same even when excluding plasmids in the analysis. This finding largely agrees with a previous study (44), which compared the number of genes in four genera (*Bacillus*, *Escherichia*, *Pseudomonas*, and Burkholderia) using a smaller data set.

**TABLE 3:** *P* values in Wilcoxon tests of P versus NP genomes of the nine genera on different subjects

Null hypothesis genus (P > NP)	*P* value for^*a*^:				
	All proteins^*b*^	Prophages^*c*^	PAIs^*d*^	VFs^*e*^	HGTs^*f*^
*Bacillus*	**1.02e−11**	0.22435869	0.856125938	*0.9999999*	0.071359632
*Burkholderia*	0.830680548	0.187561481	**0.004253136**	0.09171531	*0.9998968*
*Clostridium*	*0.997947438*	0.272854034	*0.992171529*	0.45490102	*0.999998298*
*Corynebacterium*	*0.999991211*	0.114873724	0.704693746	**2.13e−06**	*0.999999478*
*Escherichia*	**0.001769583**	**7.95e−04**	**0.005082263**	0.70368707	0.07518119
*Listeria*	0.930016112	*0.954380385*	0.795456343	**0.00744774**	0.738283882
*Mycobacterium*	*0.998296558*	0.424624239	0.816433738	**0.00127508**	*0.999929344*
*Pseudomonas*	**0.013297151**	0.130966299	*0.999881537*	**5.35e−07**	0.167064102
*Streptococcus*	**0.015876828**	0.112397253	0.538543551	0.38647105	0.356860357

a Boldface *P* values are <0.05, supporting P > NP. Italic *P* values are >0.95, supporting P < NP.

b Comparison of P and NP genomes in terms of the total number of protein-coding genes.

c Comparison of P and NP genomes in terms of % genes located in prophages = no. of prophage genes/total no. of protein-coding genes in genome.

d Comparison of P and NP genomes in terms of % genes located in PAIs = no. of PAI genes/total no. of protein-coding genes in genome.

e Comparison of P and NP genomes in terms of % VF genes = no. of VF genes/total no. of protein-coding genes in genome.

f Comparison of P and NP genomes in terms of % ORFan genes that are HGTs = no. of ORFans that are HGTs/total no. of ORFans in genome.

### Pathogens do not always have more PRGs than nonpathogens.

In Table 3, we have also compared the percentage of PRGs between P and NP genomes in each genus. (Detailed counts are available in Data Set S1.)

For prophage-carried genes, Table 3 shows that, although in *Escherichia*, pathogens tend to have more genes located in prophages than nonpathogens (44), in the other eight genera pathogens do not have more prophages than nonpathogens. For PAIs, in two genera (*Burkholderia* and *Escherichia*), the percentage of genes located in PAIs is higher in P genomes, while in two other genera (*Clostridium* and *Pseudomonas*), it is the opposite. Thus, it was inaccurate to conclude based on Table 2 that there is a higher percentage of prophages and PAIs in P genomes of all nine genera, because this is only true for *Escherichia* (Table 3), which dominated the prophage and PAI data.

For VFs, four genera (*Corynebacterium*, *Listeria*, *Mycobacterium*, and *Pseudomonas*) have a higher percentage of VF-carried genes in P than NP genomes. Lastly, for HGTs, four genera (*Burkholderia*, *Clostridium*, *Corynebacterium*, and *Mycobacterium*) have a lower percentage of ORFans derived from HGT in P than NP genomes.

Therefore, the genus-by-genus statistical tests showed that pathogens do not always have more PRGs than nonpathogens, and the observations vary between different genera.

### The percentage of PS-ORFans is always higher than that of SS-ORFans in pathogens, which is not true in nonpathogens.

**FIG 2:**
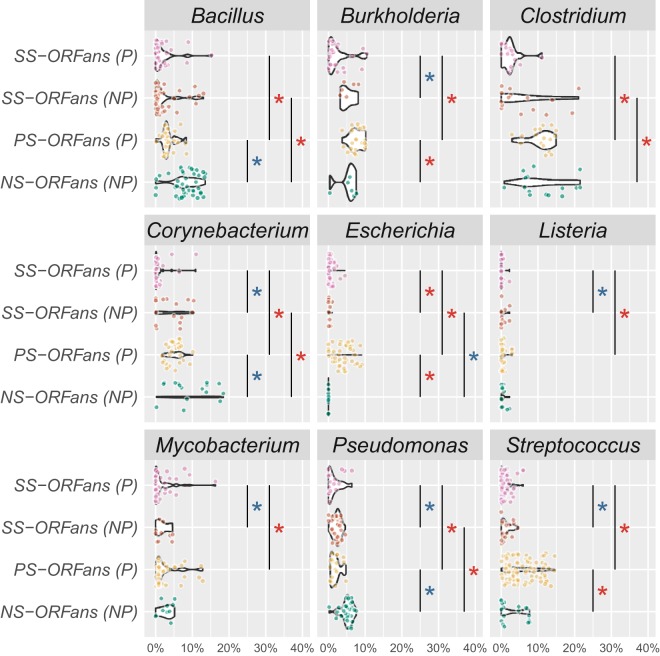
The percentages of different groups of ORFans. The violin boxplots are shown with genomes represented as dots of different colors corresponding to four groups of ORFans. For each genome, the percentages of different ORFan groups are calculated as follows: % SS-ORFans = no. of SS-ORFans/total no. of proteins in the genome. Four pairs of Wilcoxon tests were performed: (i) SS-ORFans (P) versus SS-ORFans (NP), (ii) PS-ORFans (P) versus NS-ORFans (NP), (iii) PS-ORFans (P) versus SS-ORFans (P), and (iv) NS-ORFans (NP) versus SS-ORFans (NP). Only the statistically significant differences are indicated with vertical lines and asterisks (*). Red asterisks indicate *P* value of <0.05, supporting higher SS-ORFans (P) in test pair i, higher PS-ORFans (P) in test pair ii, higher PS-ORFans (P) in test pair iii, and higher NS-ORFans (P) in test pair iv. Blue asterisks indicate the opposite.

When taking the P and NP genomes of the nine genera as a whole for comparison, a sequence of percentages was observed in Table 2: % NS-ORFans (NP) > % PS-ORFans (P) > % SS-ORFans (NP) > % SS-ORFans (P). For more accurate comparisons without bias from combining different genera, we have performed genus-by-genus statistical tests, and for each genus, four comparisons with the four groups of ORFans have been made (see Fig. 2 legend). Wilcoxon nonparametric test *P* values for these comparisons can be found in Table S2 in the supplemental material. The detailed counts of different ORFans are available in Data Set S1.

10.1128/mSystems.00290-18.2 TABLE S2 *P* values in Wilcoxon tests of different groups of ORFans in the nine genera based on the percentage of ORFans. Download Table S2, XLSX file, 0.01 MB. Copyright © 2019 Entwistle et al. 2019 Entwistle et al. This content is distributed under the terms of the Creative Commons Attribution 4.0 International license.

For the comparison SS-ORFans (P) versus SS-ORFans (NP), only in *Escherichia* was the percentage of SS-ORFans (P) significantly higher than the percentage of SS-ORFans (NP); in six genera (*Burkholderia*, *Corynebacterium*, *Listeria*, *Mycobacterium*, *Pseudomonas*, and *Streptococcus*), it is the opposite.

For the comparison PS-ORFans (P) versus NS-ORFans (NP), in three genera (*Escherichia*, *Burkholderia*, and *Streptococcus*), the percentage of PS-ORFans is significantly higher than the percentage of NS-ORFans; however, in three other genera (*Bacillus*, *Corynebacterium*, and *Pseudomonas*), it is the opposite. All of these findings suggest that nonpathogens do not necessarily have more ORFans than pathogens, because different genera behave differently.

For the comparison PS-ORFans (P) versus SS-ORFans (P), in the nine genera, the percentage of PS-ORFans is always significantly higher than the percentage of SS-ORFans. This suggests that ORFans tend to be shared by different pathogenic genomes.

However, for the comparison NS-ORFans versus SS-ORFans (NP), in four genera (*Bacillus*, *Clostridium*, *Corynebacterium*, and *Pseudomonas*), the percentage of NS-ORFans is significantly higher than the percentage of SS-ORFans, while in *Escherichia*, the percentage of NS-ORFans is significantly lower than the percentage of SS-ORFans, and in the other four genera, there is no significant difference. Therefore, unlike P genomes, NS-ORFans are not always more abundant than SS-ORFans in NP genomes.

### PS-ORFans are always more abundant than SS-ORFans in PRGs in pathogens, which is not true in nonpathogens.

We continued by comparing the percentages of different groups of ORFans in relation to the four types of PRGs (prophages in Table 4, PAIs in Table 5, VFs in Table 6, and HGTs in Table 7), which is a novel analysis of this study. For prophages, PAIs, and VFs, we first compiled a list of proteins encoded by these PRGs in each genome, and then we separated PRGs into SS-ORFans, PS-ORFans, and non-ORFans in pathogenic (P) genomes and into SS-ORFans, NS-ORFans, and non-ORFans in nonpathogenic (NP) genomes. Lastly, we calculated their percentages for Wilcoxon tests. For HGTs, non-ORFans were excluded in the Wilcoxon tests of Table 7. The detailed counts of different ORFans in different PRGs are available in Data Set S1.

**TABLE 4:** *P* values in Wilcoxon tests of different groups of ORFans in the nine genera based on the percentage of ORFans in prophages

Null hypothesis genus	*P* value for^*a*^:			
	% PS-ORFans > % SS-ORFans (P)	% NS-ORFans > % SS-ORFans (NP)	% SS-ORFans (P) > % SS-ORFans (NP)	% PS-ORFans > % NS-ORFans
*Bacillus*	**5.40e−05**	**7.26e−06**	0.394433525	0.880832477
*Burkholderia*	**7.47e−05**	*0.985259996*	*0.960055114*	**0.001097849**
*Clostridium*	**5.00e−04**	0.159456553	0.728183622	0.056349164
*Corynebacterium*	**0.040265381**	0.878883731	0.874371848	0.062736061
*Escherichia*	**4.07e−12**	0.939696489	0.094187485	**2.84e−06**
*Listeria*	**0.00300208**	0.5	*0.99621199*	0.700199577
*Mycobacterium*	**7.99e−05**	0.196771097	0.580161013	0.064978632
*Pseudomonas*	**0.02385388**	**3.81e−06**	0.943266767	0.821135897
*Streptococcus*	**1.45e−07**	*0.964384882*	0.645583377	**7.96e−05**

a Boldface *P* values are <0.05, supporting the null hypothesis in the header row. Italic *P* values are >0.95, supporting the alternative hypothesis. The percentages of the different ORFan groups in prophages are calculated as, e.g., % PS-ORFans = no. of PS-ORFans located in prophages/total no. of prophage proteins in genome.

**TABLE 5:** *P* values in Wilcoxon tests of different groups of ORFans in the nine genera based on the percentage of ORFans in PAIs

Null hypothesis genus	*P* value for^*a*^:			
	% PS-ORFans > % SS-ORFans (P)	% NS-ORFans > % SS-ORFans (NP)	% SS-ORFans (P) > % SS-ORFans (NP)	% PS-ORFans > % NS-ORFans
*Bacillus*	**1.03e−04**	**3.63e−06**	*0.963761207*	*0.999880114*
*Burkholderia*	**1.42e−07**	*0.998473601*	*0.999440835*	**2.78e−04**
*Clostridium*	**0.003776606**	0.159483531	0.838156431	0.477412277
*Corynebacterium*	**7.30e−09**	0.149975088	*0.996125288*	0.471677773
*Escherichia*	**1.95e−12**	0.926534723	0.151197704	**3.37e−06**
*Listeria*	**0.01568275**	0.453673917	0.86992928	0.366613929
*Mycobacterium*	**2.90e−04**	0.589735247	*0.977567159*	0.273832977
*Pseudomonas*	0.066440993	**0.01560009**	*0.97832476*	*0.964345358*
*Streptococcus*	**1.77e−18**	0.64218788	0.944064298	**6.14e−07**

a Boldface *P* values are <0.05, supporting the null hypothesis in the header row. Italic *P* values are >0.95, supporting the alternative hypothesis. The percentages of the different ORFan groups in PAIs are calculated as, e.g., % PS-ORFans = no. of PS-ORFans located in PAIs/total no. of PAI proteins in genome.

**TABLE 6:** *P* values in Wilcoxon tests of different groups of ORFans in the nine genera based on the percentage of ORFans of VF origin

Null hypothesis genus	*P* value for^*a*^:			
	% PS-ORFans > % SS-ORFans (P)	% NS-ORFans > % SS-ORFans (NP)	% SS-ORFans (P) > % SS-ORFans (NP)	% PS-ORFans > % NS-ORFans
*Bacillus*	**1.32e−09**	**3.66e−09**	**0.019769222**	**0.001096014**
*Burkholderia*	**5.12e−11**	0.286237787	*0.959942111*	**2.12e−04**
*Clostridium*	**1.30e−07**	0.08833981	0.928052027	**2.14e−05**
*Corynebacterium*	**2.89e−-14**	0.260592071	*0.998928193*	**4.68e−07**
*Escherichia*	**1.93e−13**	0.785348469	0.529331759	**9.92e−06**
*Listeria*^*b*^	0.5	*1*	0.274220063	0.274220063
*Mycobacterium*	**2.02e−10**	**0.01270407**	*0.959142196*	0.157008589
*Pseudomonas*	**0.01329007**	**1.95e−09**	0.62968916	*0.994360516*
*Streptococcus*	**4.19e−19**	0.060758795	*0.962078586*	**4.10e−04**

a Boldface *P* values are <0.05, supporting the null hypothesis in the header row. Italic *P* values are >0.95, supporting the alternative hypothesis. The percentages of the different ORFan groups of VF origin are calculated as, e.g., % PS-ORFans = no. of PS-ORFans of VF origin/total no. of VF proteins in genome.

b In total, only 3 ORFans of the 40 *Listeria* genomes are VFs (Data Set S1), so the *P* values for this genus are not reliable.

**TABLE 7:** *P* values in Wilcoxon tests of different groups of ORFans in the nine genera based on the percentage of ORFans of HGT origin

Null hypothesis genus	*P* value for^*a*^:			
	% PS-ORFans > % SS-ORFans (P)	% NS-ORFans > % SS-ORFans (NP)	% SS-ORFans (P) > % SS-ORFans (NP)	% PS-ORFans > % NS-ORFans
*Bacillus*	**9.69e−07**	**1.51e−12**	0.543668612	0.937425556
*Burkholderia*	**5.94e−10**	0.086742734	0.842279273	0.169238704
*Clostridium*	**4.05e−07**	**5.65e−05**	0.82867728	0.181149495
*Corynebacterium*	**6.20e−11**	**4.79e−06**	*0.986624151*	0.098310924
*Escherichia*	**1.06e−12**	0.89308897	0.659921543	**1.18e−04**
*Listeria*	**3.41e−04**	0.416257815	0.931692179	0.148137858
*Mycobacterium*	**5.47e−06**	**0.018674081**	0.889525369	0.397438826
*Pseudomonas*	**7.35e−04**	**3.96e−11**	0.17201608	0.832963242
*Streptococcus*	**1.64e−29**	0.123168263	*0.999575148*	**6.43e−07**

a Boldface *P* values are <0.05, supporting the null hypothesis in the header row. Italic *P* values are >0.95, supporting the alternative hypothesis. The percentages of the different ORFan groups of HGT origin are calculated as, e.g., % PS-ORFans = no. of horizontally transferred PS-ORFans/total no. of horizontally transferred ORFans in genome.

The most interesting observation from Tables 4 to 7 is that the percentage of PS-ORFans is significantly higher than percentage of SS-ORFans in P genomes of almost all the genera for all the four types of PRGs. (*Listeria* in Table 6 has a *P* value of 0.5, because only 1 out of the 40 *Listeria* genomes has VFs, and thus, the *P* value is not meaningful.) This also agrees with the finding made in Fig. 2 and Table S2 that in P genomes of the nine genera, the percentage of PS-ORFans is always higher than the percentage of SS-ORFans.

This finding suggests that PS-ORFans (shared by multiple P genomes) are more associated with bacterial pathogenicity than SS-ORFans (unique in each genome). In contrast, in NP genomes, the comparison of the percentages of PS-ORFans and SS-ORFans for the four types of PRGs does not show such uniformity. Particularly, for prophages and PAIs (Tables 4 and 5), most of the genera show no significant difference. This can be explained by the fact that the PRGs are not as important to P genomes as to NP genomes.

For the other three comparisons, no clear patterns are observed. The test results vary among different genera and among different PRGs.

### Overrepresented GO functions in different groups of ORFans.

GO annotation of the four groups of ORFans was performed by searching against the UniProt and Protein Data Bank (PDB) databases (see Materials and Methods). Table S3 in the supplemental material shows that overall 40% (52,383 out of 130,169) of the ORFans can be annotated with at least one Gene Ontology (GO) term. When looking at different ORFan groups, as expected, this percentage is much lower in SS-ORFans (27.37% for P genomes and 29.85% for NP genomes) than the more conserved PS-ORFans (42.73%) and NS-ORFans (46.90%).

10.1128/mSystems.00290-18.3 TABLE S3 Counts of ORFans that were assigned to GO terms in the nine genera. Download Table S3, XLSX file, 0.01 MB. Copyright © 2019 Entwistle et al. 2019 Entwistle et al. This content is distributed under the terms of the Creative Commons Attribution 4.0 International license.

To study what functions are overrepresented in ORFans, we have compared the GO annotation of our four ORFan data sets against that of a protein data set randomly selected from the entire gene content of the nine genera. A binomial test was run on each GO term to test if the ORFan count is significantly higher than the random protein count. Data Set S2 in the supplemental material provides the top-ranked GO terms that are significantly overrepresented in the four groups of ORFans. As expected, GO terms related to phages (such as DNA integration, virus tail fiber assembly, and viral genome ejection) are among the most overrepresented functions found in PS-ORFans. Interestingly, DNA integration is also in the top 10 GO terms found in the other three ORFan groups. In addition, two GO terms (DNA excision [related to DNA repair after recombination] and response to nutrient [related to extracellular stimulus]) are found in the top 10 terms for three of the four ORFan groups.

10.1128/mSystems.00290-18.6 DATA SET S2 Overrepresented GO terms in the four groups of ORFans ranked by binomial test *P* value. Download Data Set S2, XLSX file, 0.2 MB. Copyright © 2019 Entwistle et al. 2019 Entwistle et al. This content is distributed under the terms of the Creative Commons Attribution 4.0 International license.

### A database of ORFans of pathogenic bacteria.

All the ORFan data generated in this study are provided through an online database, ORFanDB (http://cys.bios.niu.edu/ORFanDB/). The website features an embedded interactive web application that allows a user to select a species and then further narrows their selection based on strain and ORFan type using a set of nested tabs. The final nested tab (“Protein Information”) reveals data about the ORFan, such as hits in PRGs, a Jbrowser instance showing the genomic neighborhood, and genome metadata curated from JGI (Joint Genomic Institute). There is also a download page from which the user can download all the data available, genus-specific data, or ORFan type-specific data. Lastly, a help page and an about page are created to provide the user with information on how to use the application.

## DISCUSSION

Previous literature has studied the four types of pathogenicity-related genes (PRGs) using comparative genomics approaches (25–27, 44). Two papers have specifically compared prophages (44) and VFs (26) between pathogens and nonpathogens. In addition, we and others have focused on developing new computational methods for the identification of ORFans in hundreds of bacterial genomes and metagenomes (2–4, 6). Despite these previous efforts, the novelty of the current work is that we have separated ORFans into four different groups, which enabled us to compare them within/between pathogens and nonpathogens of the same bacterial genus, particularly in terms of their relative abundance in the four types of PRGs.

Before this study, the previous literature had already suggested that (i) at least in some genera, P genomes are larger than NP genomes (44), (ii) ORFans are overrepresented in PAIs compared to the rest of the genome (25), and (iii) combining genomes from different genera, overall, P genomes have fewer ORFans than NP genomes (26).

Our data extended these findings. For example, for finding i, Table 3 showed that in four out of nine genera, P genomes have more genes than NP genomes, whereas in the other five genera, this is not true. For finding ii, the previous finding was extended with four groups of ORFans in Table 2, which showed the following for genes located in PAIs: % PS-ORFans (P) > % SS-ORFans (NP) ≈ % SS-ORFans (P) > % NS-ORFans (NP) ≫ % non-ORFans (P) > % non-ORFans (NP). This finding was also extended to prophages, showing the following: % PS-ORFans (P) > % SS-ORFans (P) > % NS-ORFans (NP) > % SS-ORFans (NP) ≫ % non-ORFans (P) > % non-ORFans (NP).

For finding iii, Table 2 confirmed that NP genomes have a higher overall percentage of ORFans than NP genomes, but also showed that the percentage of SS-ORFans (NP) is higher than the percentage of SS-ORFans (P), and the percentage of NS-ORFans (NP) is higher than the percentage of PS-ORFans (P). However, we argued that an unbiased genus-by-genus comparison was required to obtain a more accurate result. When comparing them in each genus (Fig. 2 and Table S2), the percentages of NS-ORFans (NP) and SS-ORFans (NP) were no longer always higher than those of PS-ORFans (P) and SS-ORFans (P), respectively. For example, in *Escherichia*, the percentage of PS-ORFans (P) was significantly higher than that of NS-ORFans (NP) and the percentage of SS-ORFans (P) was significantly higher than that of SS-ORFans (NP).

The most significant findings of this study are that in pathogens of the nine genera, the percentage of PS-ORFans was consistently higher than that of SS-ORFans (Fig. 2 and Table S2), and the percentage of PS-ORFans annotated to be PRGs (all the four types) was also consistently higher than that of SS-ORFans (Tables 4 to 7). These findings were even more intriguing when seeing in nonpathogens of the nine genera that such a strong and uniform pattern (i.e., % NS-ORFans > % SS-ORFans) across all the nine genera did not exist.

**FIG 3:**
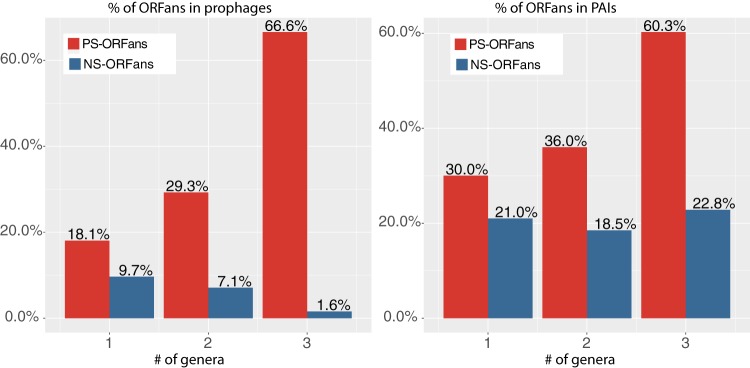
More conserved PS-ORFans (but not NS-ORFans) are more likely to be found in prophages and PAIs. The *x* axis is the number of genera in which an ORFan has blastp hits. (The number is 1 for an ORFan restricted to its own genus.) The *y* axis is the percentage of ORFans (e.g., the number of ORFans located in prophages divided by the number of ORFans). The detailed numbers are available in Table S4.

To add even more support for these findings, we have run “all versus all” blastp search on the 56,196 PS-ORFan and 39,437 NS-ORFan data sets (Table 2) separately. Then we counted how many genera each query ORFan had hits in. In total, 2,437 (4.34%) PS-ORFans and 2,088 (5.29%) NS-ORFans also have blastp hits in other genera than their self-genus. After grouping ORFans based on the number of genera (ORFan conservation), we plotted the percentages of each group matching prophages and PAIs and observed a positive correlation for PS-ORFans but not for NS-ORFans (Fig. 3). We also did the same for VFs and HGTs (see Table S4 in the supplemental material). VFs showed a similar pattern, but the numbers were too small to be significant. HGTs had positive correlations in both PS-ORFans and NS-ORFans. Overall, this further suggests that the more conserved PS-ORFans (found in more genera) are, the more likely they are pathogenicity related. In contrast, this is not true for NS-ORFans—at least in prophages and PAIs.

10.1128/mSystems.00290-18.4 TABLE S4 PS-ORFans and NS-ORFans that have hits in other genera. Download Table S4, XLSX file, 0.01 MB. Copyright © 2019 Entwistle et al. 2019 Entwistle et al. This content is distributed under the terms of the Creative Commons Attribution 4.0 International license.

From the evolutionary selection perspective, new genes from phages, distant bacteria, PAIs, and other mobile genetic elements can constantly enter the host genome through horizontal gene transfer; however, these new genes have to go through the natural selection process, where only those providing selective advantage to their bacterial hosts (i.e., pathogenicity) are eventually fixed in the pathogen population (e.g., found in multiple pathogenic genomes of the same genus).

It should be mentioned that such an HGT selection model works for any genes and any biological processes in any genomes. Notably, in nonpathogens, we also observed a significant percentage of ORFans and PRGs (Table 2). However, the selection of PRGs and ORFans in nonpathogens may not be as strong and universal as in pathogens.

These findings strongly suggest that the PS-ORFans that are shared by multiple pathogens have a higher success rate to transform a nonpathogen to a pathogen compared to SS-ORFans. Therefore, PS-ORFans should be considered better targets to identify novel PRGs and to develop diagnostic/therapeutic drugs.

Lastly, other than ORFans that originated through horizontal gene transfer (gene gain) from phages or other bacteria, there are other important factors that can also account for bacterial pathogenicity, such as gene loss due to genome reduction (i.e., smaller P genomes), modification of the core genome (non-ORFans) with single nucleotide polymorphisms (SNPs), indels, and recombinations (42, 43, 45). Although not a focus of this study, some of these factors such as SNPs found in PRGs of non-ORFans may be a more plausible reason for infectious disease outbreaks, which usually happen in a relatively short evolutionary time scale, as revealed by the numerous recent whole-genome shotgun sequencing efforts for genomic epidemiology studies (e.g., reviewed in references 46, 47, and 48).

## MATERIALS AND METHODS

### Genome data.

In total, 6,005 completely sequenced and assembled bacterial genomes were downloaded from the RefSeq database (ftp://ftp.ncbi.nih.gov/genomes/refseq/bacteria) as of August 2017, denoted as Bacteria-DB.

A list of bacterial genomes at http://www.pathogenomics.sfu.ca/pathogen-associated/2014/ was manually curated and classified into pathogen (P) and nonpathogen (NP) groups by the Brinkman lab (26). As this list was from an older version of the RefSeq database, there were a smaller number of genomes curated and available in the above web link than the Bacteria-DB we used. The 2,864 GenBank accession numbers (ACs) of these genomes were used to extract their RefSeq data files (genomic fna, protein faa, etc.) from the Bacteria-DB. Out of the 2,864 ACs, 2,479 were found in Bacteria-DB. Nine genera with >5 pathogenic and >5 nonpathogenic genomes (in total, 505 genomes) were kept for further analyses.

### ORFan identification.

As shown in Fig. 1, for each bacterial genus, we used all of its genomes (P and NP) to make a combined proteome (all proteins of a genome). We then ran an “all versus all” blastp search (E value of <0.01) using DIAMOND (49), and based on the search result, we classified proteins of each genome into the following:

1. SS-ORFans: strain-specific ORFans, defined as proteins with DIAMOND hits restricted to the query genome (two groups of SS-ORFans: those from P and those from NP)
2. PS-ORFans (only in P): pathogen-specific ORFans, defined as proteins with DIAMOND hits restricted to ≥2 pathogenic genomes
3. NS-ORFans (only in NP): non-pathogen-specific ORFans, defined as proteins with DIAMOND hits restricted to ≥2 non-pathogenic genomes
4. Non-ORFans: defined as the rest of proteins in the genomes

### PRGs.

Four types of genes were identified in the 505 genomes: prophage genes, PAI genes, VF genes, and HGT genes.

The genomic locations of ORFans were compared to the genomic locations of prophages in the PHASTER database (50) and to the genomic locations of PAIs in the IslandViewer database (51). The ORFan genes in prophages and PAIs were then classified into SS-ORFans, PS-ORFans, and NS-ORFans groups.

To determine if an ORFan is a virulence factor (VF) gene, ORFan sequences were blastp searched against the VFDB (52) using DIAMOND (E value of <1e−5).

Horizontally transferred (HGT) genes were identified as proteins having limited blastp hits in taxonomically close (genus-level) genomes but more hits in taxonomically distant (order-level) genomes. To determine if an ORFan is horizontally transferred, ORFan sequences were blastp searched against the protein sequences of the Bacteria-DB (6,005 genomes of various taxonomic phyla) using DIAMOND (E value of <1e−5). We defined an ORFan to be horizontally transferred if it has very few blastp homologs within the studied genus, but has blastp homologs in other taxonomic orders. Specifically, the DIAMOND result was filtered to remove all hits of the same genus as the ORFan query. Then the taxonomic lineages of the remaining hits were examined. If the ORFan has all its remaining hits from different taxonomic orders (two levels up from genus in the taxonomy hierarchy), it means that the ORFan does not have blastp hits in other genomes of the same genus than those used for ORFan identification, but has hits in genomes of more distant orders. This is evidence of gene transfer from distant organisms, and such ORFans were retrieved as HGTs.

For example, a PS-ORFan protein, WP_001086421.1, from Escherichia coli APEC O1 (GCF_000014845) has a small number of blastp hits within the *Escherichia* genus (all hits are from pathogenic genomes) and no other hits within the Enterobacterales order. However, it has numerous hits in other orders of the Gammaproteobacteria class and orders of other bacterial phyla. Such atypical taxonomic distribution of WP_001086421.1’s blastp hits can be explained either by HGT from distant organisms into pathogens of the *Escherichia* genus or by massive gene loss within the *Enterobacterales* order. As the *Enterobacterales* order is one of the most sequenced bacterial orders (thousands of genomes in Bacteria-DB), the chance of massive and independent gene loss is much smaller than the chance of recent HGT. This is true for all the genomes of the nine genera, for they are all from well-represented orders in the genome database.

### Functional annotation of ORFans.

We modified a workflow reported in reference 3 to annotate ORFans for Gene Ontology functional descriptions. DIAMOND was used to compare all the ORFans to the UniProt database. The best hit of each ORFan was kept if the alignment identity was ≥80% and the E value was ≤0.01. The GO terms of the UniProt hits were then assigned to the ORFans by parsing the UniProt ID mapping file downloaded from the UniProt ftp site. In total, 39,330 ORFans were annotated with GO using UniProt2GO.

ORFans that were not annotated by UniProt2GO were then compared to the PDB70 database using the more sensitive profile-based tool hhsearch (53). The results were parsed to keep the best hit if the probability threshold was ≥80% and the E value was ≤1. The GO terms of the PDB hits were then assigned to the ORFans by parsing the PDB2GO mapping file downloaded from the GOA (GO annotation) ftp site. In total, 13,053 ORFans were annotated with GO using PDB2GO. Altogether, 52,383 ORFans were mapped to GO terms.

For GO enrichment analysis, 100,000 proteins were randomly selected from the nine genera, and subjected to the same workflow to be mapped to GO terms. The R function binom.test was used to compare the number of ORFans with a specific GO term (limited to the 5th level of GO terms from BP [biological process] and MF [molecular function] categories) to the number of random genes with the same GO term. P.adjust in R was used to adjust for multiple comparisons.

### Data availability.

The data from this study were organized into a MySQL database. A web application was written in R, using primarily the Shiny package, to provide a user interface to explore these data. Shiny Server was used to host the publicly available website, ORFanDB, in which all of the ORFan data have been made available (http://cys.bios.niu.edu/ORFanDB/).
